# The Influence of the Diameter of Orthodontic Mini-Implants on Primary Stability: Bending Tests—An In Vitro Study

**DOI:** 10.3390/ma17133149

**Published:** 2024-06-27

**Authors:** Catarina Sousa-Santos, Sofia Sousa-Santos, Joana Mendes, Carlos Coelho, Carlos Aroso, Primavera Sousa-Santos, José Manuel Mendes

**Affiliations:** 1 Department of Dental Sciences, University Institute of Health Sciences (IUCS), CESPU, 4585-116 Gandra, Portugal; catarina.sousa.santos@hotmail.com; 2 Oral Pathology and Rehabilitation Research Unit (UNIPRO), University Institute of Health Sciences (IUCS), CESPU, Rua Central da Gandra 1317, 4585-116 Gandra, Portugal; sofia.sousa.santos@hotmail.com (S.S.-S.); carlos.coelho@iucs.cespu.pt (C.C.); carlos.ribeiro@iucs.cespu.pt (C.A.); primavera.santos@iucs.cespu.pt (P.S.-S.); jose.mendes@iucs.cespu.pt (J.M.M.)

**Keywords:** miniscrews, orthodontic Mini-Implants, deformation, primary stability

## Abstract

Orthodontic Mini-Implants have a high success rate, but it is crucial to assess the load that they bear in order to maintain their primary stability. Increasing the diameter can improve this stability, but there are limitations due to the proximity of the tooth roots. To avoid damage, smaller diameters are used, which can decrease resistance and cause permanent deformations. Objective: The objective of this study is to evaluate the influence of the diameter of Mini-Implants through bending force tests, taking into account primary stability after one and two insertions. Methods: Here, 40 Ti6AI4V alloy Mini-Implants of two different brands and diameters were divided into eight groups, half of which received one insertion in the artificial bone, and the rest received two. All were subjected to a constant bending force using an INSTRON-Electropuls E10000LT (Norwood, MA, USA) until fracture. Results: The smaller-diameter Mini-Implants were less resistant to fracture, but both were able to withstand the necessary loads produced by orthodontic movements. As for the inserts, there were no statistically significant differences. Conclusions: There is an advantage to using 1.6 mm Mini-Implants over 2.0 mm ones, as a smaller diameter does not lead to fracture due to the forces used in orthodontic treatment. Having one or two inserts did not have a statistically significant effect.

## 1. Introduction

Orthodontic treatment requires stable anchorage, and some traditional orthodontic appliances depend considerably on the patient’s cooperation in reinforcing this anchorage. In order to improve this factor, Mini-Implants (MIs) have emerged as an alternative that is independent of the patient’s cooperation.

The use of MIs for skeletal anchorage has increased in orthodontic practice, making treatment more efficient and faster since it does not depend directly on the patient’s cooperation [1,2]. The use of MIs as an aid in orthodontic treatment has had a success rate from 75% to 90% [3]. One of the crucial points when placing these devices is determining the torque required and assessing the load they can bear in order to preserve primary stability and absolute anchorage [3,4]. Even with a high success rate, the reattachment of MIs is a common procedure. The need for this procedure may arise due to anatomical limitations or the loss of primary stability.

Stability is achieved through mechanical retention since MIs do not undergo the process of osseointegration. Due to their great advantages, these devices have several indications, including intrusions, extrusions, medialisation, dental distalisations and the treatment of open and deep bites [4,5]. Primary stability (obtained immediately after placement in the bone) is crucial for MIs’ success [4,6,7]. These devices can be applied to practically the entire oral cavity and used with different types of forces [6]. Improving primary stability can be achieved by increasing the diameter and length of Mis, which translates to better performance. However, this increase is limited by the proximity of adjacent teeth roots and the risk of contact [3]. To ensure MIs’ good primary stability, several factors need to be considered, such as the type of the bone (cortical and trabecular), the characteristics of the Mini-Implant (MI) (diameter, length and shape), its position (placement angle), the condition of the gingival tissue around the MI, the patient’s age (considering that the quantity and quality of bone increases with age) and the force applied. If a load is applied to the MI without it having sufficient stability, loss of stability may occur [4,7,8,9]. Studies indicate that titanium alloy MIs remain stable with forces of up to 250 g and are more effective when subjected to immediate forces [8].

The literature points out that, in orthodontic practice, the most commonly used MIs are those made of the titanium alloy Ti6Al4V due to their greater resistance to corrosion and greater biocompatibility, are less likely to be rejected when placed on patients, leading to an increase in MIs performance [10]. The minimum recommended length for MIs is around 6 mm and usually goes up to 10 mm, and the diameter usually varies between 1.3 mm and 2.0 mm [11].

Although one of the main advantages of MIs is the low risk of complications, it is possible for these to occur. To minimise the risk of injury to adjacent anatomical structures, smaller diameters are used. However, a significant issue is that reducing the diameter of MIs results in a decrease in resistance to both the maximum fracture torque and the amount of load supported, which can lead to permanent deformation. This happens because decreasing the diameter leads to less resistance to the load it can bear, which means that smaller diameters will be less resistant to bending forces, which can compromise primary stability [10].

Therefore, the first aim of this study was to evaluate the influence of the diameter and brand of MIs on their resistance through mechanical tests of bending forces, thus evaluating MIs’ primary stability. The second objective was to assess primary stability after one and two insertions. Two brands used in orthodontic practice were evaluated.

## 2. Materials and Methods

### 2.1. Materials

All materials and chemicals were used in accordance with the manufacturers’ standards. This study used two brands of Mini-Implants (MIs), one of which was Fatscrew (Fts) from Air Orthodontics^®^ (Barcelona, Spain). The other brand of MIs was a white brand (MB) produced by Worldtrade-center^®^ (Beijing, China) marketed by eBay^®^ (San Jose, CA, USA). These MIs were inserted into an artificial bone with characteristics similar to those of the human jawbone—Sawbones^®^ (Sawbone Europa AB, Malmö, Sweden).

### 2.2. Methods

A standard laboratory protocol was established and applied to test all selected samples at the Laboratory of Investigation in Oral Rehabilitation and Prosthodontics, UNIPRO-Oral Pathology and Rehabilitation Research Unit, University Institute of Health Sciences (IUCS), CESPU, Gandra, Portugal.

#### 2.2.1. Sample Preparation

Forty Ti6AI4V alloy (grade V) self-drilling MIs were used in this study—twenty Fatscrew (Fts) brand MIs from Air Orthodontics^®^ and twenty white brand (MB) MIs from Worldtrade-center^®^. Regarding each brand, 10 MIs were 1.6 mm in diameter, and the remaining 10 were 2.0 mm in diameter. The 40 MIs were divided into eight groups of 5 MIs each, with four groups having only one placement in the artificial bone and the remaining four groups having two insertions in the bone: Group 1 (5 MI Fts of 1.6 mm Ø, placed once in the artificial bone), Group 2 (5 MI Fts of 1.6 mm Ø, placed twice in the artificial bone), Group 3 (5 MI Fts of 2.0 mm Ø, placed once in the artificial bone) Group 4 (5 MI Fts of 2.0 mm Ø, placed twice in the artificial bone), Group 5 (5 MB MIs of 1.6 mm Ø, placed once in the artificial bone), Group 6 (5 MB MIs of 1.6 mm Ø, placed twice in the artificial bone), Group 7 (5 MB MIs of 2.0 mm Ø, placed once in the artificial bone) and Group 8 (5 MB MIs of 2.0 mm Ø, placed twice in the artificial bone).

#### 2.2.2. Elaboration of Artificial Bone Blocks

The MIs were placed in artificial bone with characteristics similar to those of a human jaw bone. The material used was Sawbones^®^ (Sawbone Europa AB, Malmö, Sweden). Sawbones’ epoxy formulation is filled with short glass fibres and used to simulate cortical bone for structural testing. This simulated cortical bone has a density (2.0 g/cc), fracture toughness (6.0 MPa), Tensile Strength (150 MPa), Tensile Modulus (20 GPa), Flexural Modulus (20 GPa), Flexural Strength (225 MPa) and hardness all similar to cadaveric cortical bone. This material is the cortical bone for all our absolute bones. It is a grey/green colour. Cellular, rigid polyurethane foam has larger pores to resemble cancellous bone. There are different types of density to be chosen, and it presents an off-white colour [12].

In order to better represent the human jawbone, a 2 mm-thick sheet of 4th-generation fibre-filled epoxy with the following specifications was used in the cortical bone layer: short fibre filler epoxy, the direction of fibre parallel to width (120 mm), density ± 2.5%.

**Figure 1:**
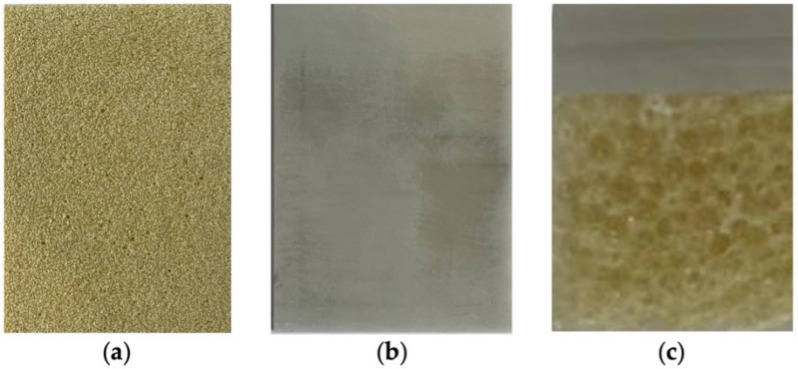
Artificial bone blocks: (**a**) spongy bone; (**b**) cellular rigid polyurethane foam; (**c**) bone simulation block.

To represent the cancellous bone, we used a rigid cellular foam block of 20 PCF (0.32 g/cm^3^) with a thickness of 10 mm (Figure 1a) with the following specifications: cellular, rigid polyurethane foam, thickness parallel to the direction of rise, density ± 10%. The epoxy sheet was coupled to the rigid cellular foam block with cyanoacrylate, as indicated by Sawbones^®^, Figure 1b. This bone material was divided into 1.5 × 1.5 cm fragments (Figure 1c).

#### 2.2.3. Insertion into Artificial Bone

**Figure 2:**
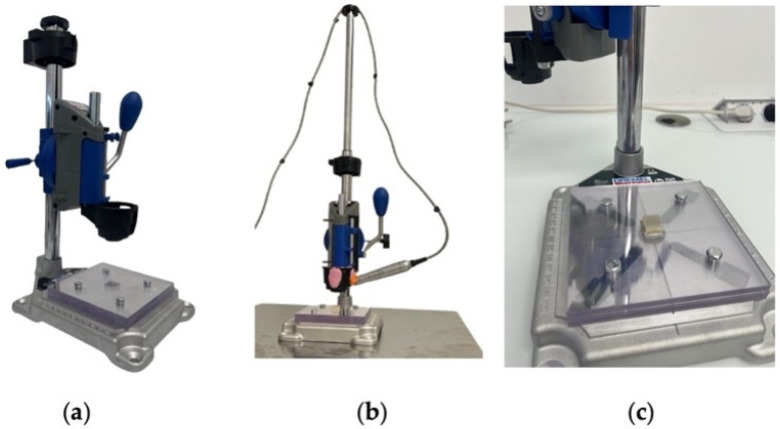
Preparation of test specimens. (**a**) Drilling support machine; (**b**) fixing the motor; (**c**) calibration when placing the MIs.

The MIs were placed using a drill stand machine, Figure 2a, to which an Implantmed^®^ Plus SI-1023 micromotor with an S-NW-W&H wireless foot pedal (REF: 30288000) made in Austria, was attached.

In order to place all the MIs, the micromotor was calibrated at 50 rpm with a maximum torque of 60 N/cm. Zetalabor and R&S Turbocclusion silicone, shown in pink and orange in Figure 2b, was used to ensure that the counter-angle of the micromotor remained firm during MI placement and did not move.

For the MIs that were placed twice in the artificial bone, the second was placed next to the first one. In this way, it was possible to retrace their placement so as to avoid operator errors, which often occur. In order to ensure MIs’ uniform placement in the artificial bone, the centres of all the bone blocks were determined (Figure 2c), and the MIs were then placed in a holder so that they were fixed in the same position as in the drilling machine. This ensured that there was no variability in the placement of the MIs on the artificial bone so that in later bending force tests, no operator error would occur.

#### 2.2.4. Compression Test to Measure the Fracture Resistance of Different Mini-Implants

The 40 MIs were subjected to a single-load bending force at a constant speed of 10 mm/min on the INSTRON^®^ Electropuls E10000 LT universal testing machine (Norwood, MA, USA). To provide fixed support for the sample and ensure compatibility with the INSTRON^®^ machine, the artificial bone material was embedded in a 2.0 × 2.0 cm metal support with Probase Cold Self-Curing Acrylic from Vivadent^®^ (Madrid, Spain). This facilitated its connection to the Electropuls E10000 LT testing machine, which is a dynamic fatigue testing machine with a linear dynamic capacity of 10 KN, a linear static capacity of 7 KN, a linear stroke of 60 mm and a torque capacity of 100 Nm, which allows static and dynamic axial and torsional tests in accordance with the ISO 7500-1 standard [13]. It has an accredited calibration force of up to 5 meganewtons in accordance with ISO 7500-1 and ASTM E4 [14].

**Figure 3:**
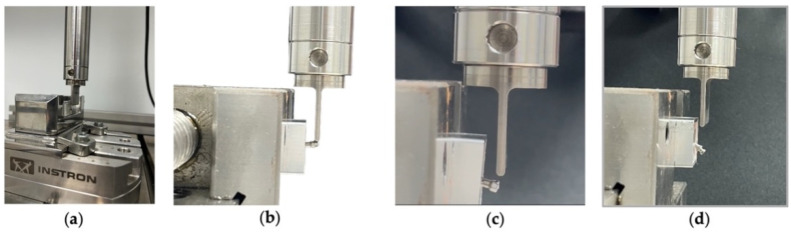
Fracture toughness tests of the MIs. (**a**) Fitting the samples to the Instron^®^; (**b**) initiation of loading to fracture; (**c**) deformation to loading; (**d**) complete fracture of the MIs.

The fracture test was carried out via compressive, which was applied to the neck of the Mini-Implant (MI) and coupled to the load cell of the testing machine. The fracture was notified by an audible click and confirmed by a sharp drop in the load-deflection curve; the test results were recorded using Bluehill Calculation Reference Software (Instron^®^, Norwood, MA, USA), which facilitated the definition and execution of tests and data acquisition. Subsequently, all values were statistically analysed. The loads required for fracture were recorded in Newtons (N). The bending force depended on the strength of the MIs and was detected by the load cell. The points of deformation initiation and fracture, determined by the load cell, were considered the key points of this test, as shown in Figure 3a–d. All this information was transferred directly to the computer connected to the machine.

#### 2.2.5. Statistical Analysis

Data were analysed with R software, version 4.3.2 [15]. Three-way ANOVAs (3-way ANOVAs) with type II sum of squares were used [16]. Type II sum of squares (SS) was chosen in place of type III sum of squares because it involves comparing the change in SS to a model with all other effects of equal or lower order (e.g., three-way interactions, two-way interactions and main effects), and Type III SS compares SS with a model containing all other effects (regardless of the order). We calculated and assessed the main 2-way and 3-way effects of brand, insertions and diameter for use as fixed effects. Generalised eta squared (η^2^_g_) was calculated to measure the effect sizes of the main 2-way and 3-way effects, according to Cohen’s [17] benchmarks for small (0.01), medium (0.06) and large (0.14).

Residual QQ plots and Shapiro–Wilk tests were used to assess the residuals’ normality. Levene’s test was used to test for variance homoscedasticity. For both tests, the null was not rejected for *p* > 0.05. Statistical significance was determined at 5%.

## 3. Results

### 3.1. Primary Stability Loss

Table 1, Table 2, Table 3 and Table 4 show the main effects (Table 1), two-way effects (Table 2 and Table 3) and three-way effects of brand, insertions and diameter in force (N) at loss of primary stability (Table 4).

The main effects of brand and diameter (Table 1) were statistically significant—F(_1,32)_ = 9.06, *p* = 0.005, η^2^_g_ = 0.22 and F(_1,32)_ = 201.59, *p* < 0.001, η^2^_g_ = 0.86, respectively, both with high effect size. The use of Fatscrew (Fts) and a diameter of 2.0 mm led to increased force (N) at loss of primary stability. Having one or two insertions did not show a significant main effect, *p* = 0.207.

Brand’s interaction with diameter (Table 2) was statistically significant, F_(1,32)_ = 35.57, *p* < 0.001, η^2^_g_ = 0.53, with a high effect size. Using a diameter of 2.0 mm led to an adjusted mean of 178.75 for Fatscrew (Fts) and 198.01 for white label, contradicting the results for the main effects. This was due to the brand effect of a lower diameter (1.6 mm), whereby the adjusted mean was 125.09 for Fatscrew (Fts) and 66.61 for the white label (*p* < 0.001).

Brand’s interaction with insertions (Table 3) was statistically significant, F_(1,32)_ = 5.25, *p* = 0.029, η^2^_g_ = 0.14, with a high effect size. Differences between brands were statistically different with two insertions (*p* < 0.001), with adjusted means of 163.58 for Fatscrew (Fts) and 129.04 for white label, but no statistical difference for one insertion.

Three-way effects (Table 4) were not statistically significant. The highest results for force (N) at the loss of primary stability were found for the white label at 2.0 mm diameter, closely followed by Fatscrew (Fts) at 2.0 mm, but only with two insertions.

**Figure 4:**
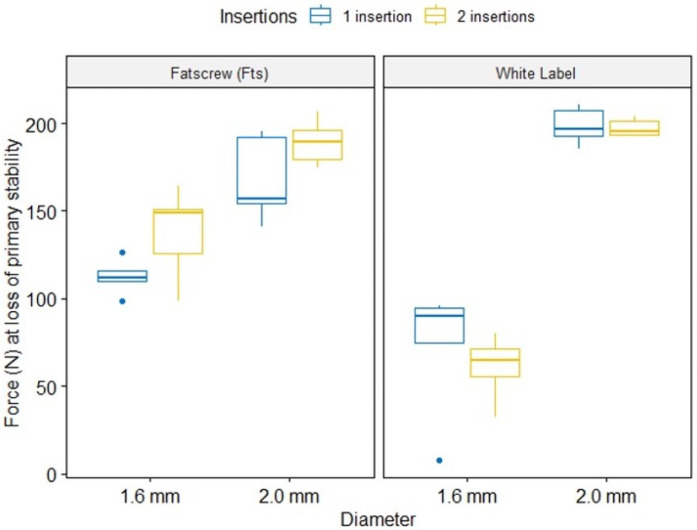
Boxplots for 3-way interactions of force (N) in the loss of primary stability model.

**Figure 5:**
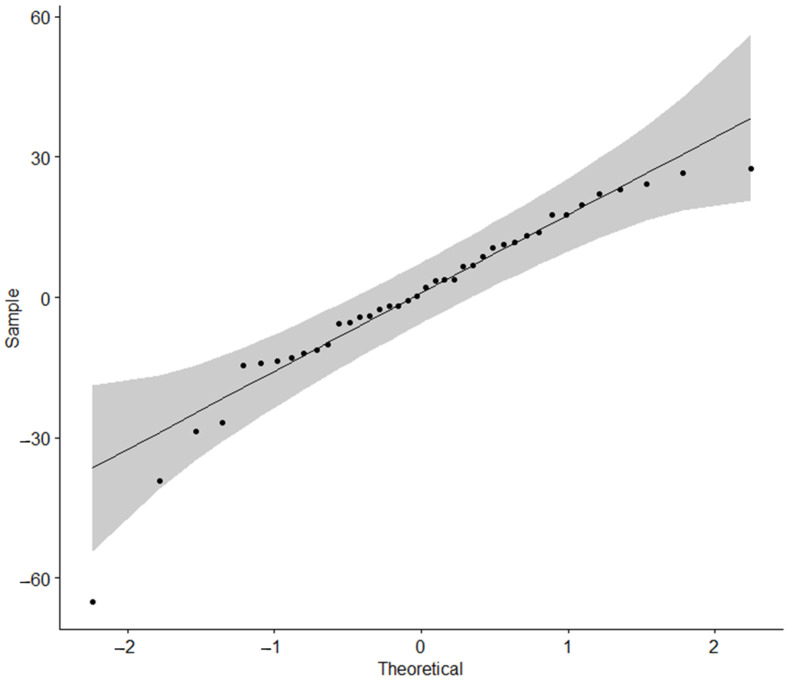
QQ plot for residuals of force (N) in the loss of primary stability model.

Figure 4 shows boxplots for 3-way interactions. Fatscrew adjusted means were very close to the ones exhibited on the white label for 2.0 mm. For 1.6 mm, mean Force (N) was higher in Fatscrew (Fts), particularly for 2 insertions, whilst in white label, this was the lowest result of all. Figure 5 shows that the normality assumption for residuals was met, with all points falling within 95% normal bounds. The result of the complementary Shapiro–Wilk test was *p* > 0.05. Variances were homoscedastic, with F_(7,15)_ = 0.74, *p* = 0.638.

### 3.2. Fracture

Table 5, Table 6, Table 7 and Table 8 show the main effects (Table 5), two-way effects (Table 6 and Table 7) and three-way effects of brand, insertions and diameter on force (N) at fracture (Table 8).

The main effects of brand and diameter (Table 5) were statistically significant—F_(1,32)_ = 9.39, *p* < 0.001, η^2^_g_ = 0.50 and F(_1,32)_ = 402.42, *p* < 0.001, η^2^_g_ = 0.93, respectively, both with a high effect size. Fatscrew (Fts) and a diameter of 2.0 mm led to increased force (N) at fracture. Having one or two insertions did not lead to a significant main effect, *p* = 0.598.

Brand’s interaction with diameter (Table 6) was statistically significant, F_(1,32)_ = 49.32, *p* < 0.001, η^2^_g_ = 0.61, with a high effect size. A diameter of 2.0 mm led to an adjusted mean of 202.15 for Fatscrew (Fts) and 209.13 for white label, with these being very similar results that do not explain the differences found in the main effect of the brand. This was due to the effect of the brand with a lower diameter (1.6 mm), where the adjusted means were 138.08 for Fatscrew (Fts) and 76.03 for the white label (*p* < 0.001).

Brand’s interaction with insertions (Table 7) was statistically significant—F_(1,32)_ = 6.87, *p* = 0.013, η^2^_g_ = 0.18, with a high effect size. Differences between brands were statistically different for two insertions (*p* < 0.001), where the adjusted means were 177.76 for Fatscrew (Fts) and 137.45 for white label, and for one insertion (*p* < 0.001), the adjusted means were 162.36 for Fatscrew (Fts) and 147.71 for white label.

Three-way effects (Table 8) were statistically significant—F_(1,32)_ = 6.87, *p* = 0.013, η^2^_g_ = 0.18, with a high effect size. The three-way interactions showed that the highest results for force (N) at fracture were produced by white label products with a 2.0 mm diameter, closely followed by Fatscrew (Fts) with 2.0 mm. The white-label brand showed the worst performance of all, with a 1.6 mm diameter.

**Figure 6:**
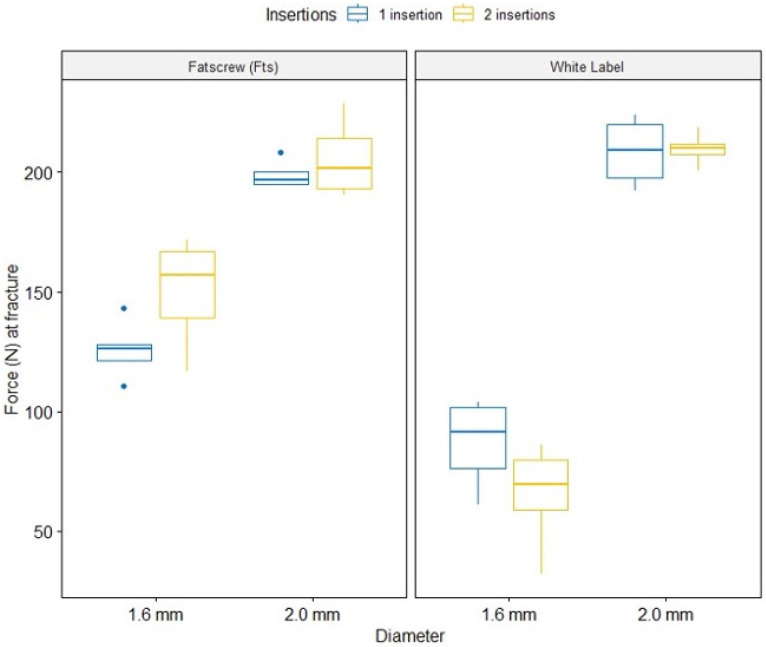
Boxplots for three-way interactions of force (N) at fracture.

**Figure 7:**
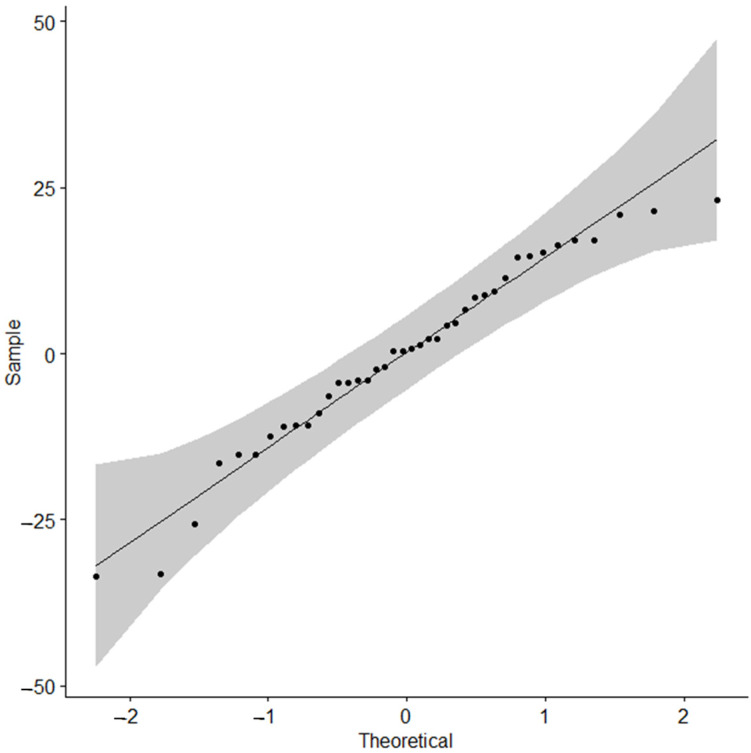
QQ plot for residuals of force (N) at fracture.

Figure 6 shows boxplots for the three-way interactions. Figure 7 shows that the residual normality assumption was met, with all points falling within 95% normal bounds. The complementary Shapiro–Wilk test result was significant at *p* > 0.05. Variances were homoscedastic, with F_(7,15)_ = 0.32, *p* = 0.356.

## 4. Discussion

The use of Mini-Implants (MIs) for temporary skeletal anchorage (TAD) has increased in orthodontic practice, making treatment faster and more efficient [1,2]. However, there are studies that report a failure rate of approximately 10–15% [3,18]. In order to reduce this failure rate, several parameters need to be assessed and analysed. The main one is the preservation of the primary stability of the MIs, for which it is necessary to study the insertion and removal torque or the load they can bear through assessment of the horizontal forces applied to them [3,4,6]. During orthodontic treatment, the MIs are subjected to forces perpendicular to their axes, so in order to test the MIs in our study, horizontal resistance tests were carried out [16].

According to the American Society for Testing and Materials (ASTM), rigid polyurethane foam is an ideal material for testing MIs as well as other medical equipment (ASTM F1839-08(2012)) [19]. Although artificial foams have limitations and do not fully represent real human jawbone, they are widely used in biomechanical tests, simulation and the evaluation of dental implants [20,21,22]. According to numerous articles, the Sawbones are one of the bones with the most similar characteristics to the human bone [6,7,18,23]. In order to be able to represent the human jawbone, two artificial bones with different characteristics were needed to represent the spongy part as well as the cortical jawbone, so we were able to maximise the similarities to the human bone. In our study, all the MIs were placed in a Sawbones^®^ artificial bone block to better represent human bone, a procedure used in several similar studies [6,7,18,23]. In this study, a 2 mm-thick sheet of fibre-filled epoxy was used to represent the cortical bone and a 10 mm-thick rigid block of 20 PCF (0.32 g/cm^3^) cellular foam was used to represent the cancellous bone, a technique that was used by Hergel et al. when they evaluated primary stability through various tests and the effects of sterilisation [6].

It may be necessary to replace the MIs due to factors such as anatomical limitations or loss of primary stability. In terms of patient safety and ethics, MIs can only be placed in the same patient, and the same Mini-Implant (MI) cannot be used in several patients. Another aspect to be considered is the economic aspect, whereby one must try to minimise the medical costs associated with orthodontic treatment. Kim et al. [18] and Hergel et al. [6] concluded in their studies that MIs can be replaced twice, especially in cases of failure.

Many studies have shown that one of the characteristics of MIs that improve primary stability, as well as fracture resistance, is the diameter of the MI chosen [4,7,10,24]. However, it is important to note that this increase is restricted by the proximity of adjacent tooth roots [3]. The average inter-radicular space typically ranges from 2.5 mm to 3.5 mm, which means that using MIs with diameters larger than 2 mm poses a risk of unintended root contact [25]. Regarding the maximum diameter, microfractures are especially common when using MIs with a diameter of 2 mm. Thus, diameters of 1.5 mm and 1.6 mm offer a good balance between MI durability and minimising cortical damage [25,26]. It is crucial to be cautious of the load applied, as the lack of sufficient stability in MIs can result in permanent dislocations [4,7,8,9]. Wilmes et al. [7] observed that the diameter of the MIs was significantly associated with their stability. The authors reported that MIs with a diameter of 2.0 mm achieved greater primary stability compared to those with a diameter of 1.6 mm. In this study, only torsional force tests were used. Barros et al. [10], in addition to torsional strength, evaluated flexural strength, showing that this was significantly influenced by the diameter of the MI. The authors concluded that the diameter influenced 83.5% of the total variation in flexural strength. This was also confirmed in the study of Haghigh et al. [24], where diameter played an important role in reducing tension and displacement compared to length. Haghigh et al. [24] stated that there was a 53% contribution of diameter and that it is advisable to increase diameter and length first if stability is at risk. Chatzigianni et al. [4] determined that at low perpendicular forces (0.5 N), there were no significant differences in displacement according to MI diameter. At higher forces (2.5 N), 2.0 mm diameter MIs moved significantly less than 1.5 mm diameter IMPs. It was also concluded that length and diameter had a statistically significant influence at a force above 1 N.

In our study, the 2.0 mm diameter MIs (groups G3, G4, G7 and G8) showed statistically significant differences in terms of greater resistance to bending force, manifesting in both fracture and displacement, compared to the 1.6 mm (G1, G2, G5 and G6). For the average fracture force, the 2.0 mm MIs showed a force of 205.64 N, and the 1.6 mm MIs only had a force of 107.06 N. The fracture was detected by an audible pop during the investigation and confirmed by a sharp drop in the load-deflection curve.

According to Hergel et al., the repeated use of MIs can compromise their stability and performance [6]; however, in our study, no significant differences were found in the stability and performance of MIs with repeated insertions compared to single use, indicating that there is less of a problem with using them twice.

As for placement in the bone, with one or two insertions, there were no statistically significant differences, either in the loss of primary stability or in the fracture, with the 1.6 mm MIs placed once in the bone (groups G1 and G5) showing less resistance compared to the 1.6 mm MIs placed twice in the bone (groups G2 and G6). As such, the null hypothesis that there is no statistically significant difference in resistance to bending forces depending on the number of insertions in the bone using 1.6 mm MIs is accepted.

The same is true for the 2.0 mm MIs. Those placed once in the bone (groups G3 and G7) showed less resistance compared to those placed twice (groups G4 and G8). Thus, the null hypothesis that there is no statistically significant difference in resistance to bending force depending on the number of insertions in the bone using the 2.0 mm MIs is accepted. These results were also confirmed by Hergel et al. [6], who concluded that there was no statistically significant difference between MIs used twice and new MIs in terms of primary stability.

In our study, even though the results show statistically significant differences depending on the diameters of the MIs, MIs with a diameter of 1.6 mm can be used for temporary anchorage in orthodontic treatment regardless of the type of movement to be carried out. This is because the force required ranges from 10 g for dental intrusion to 120 g for individual tooth movements (body movement, translation), which correspond to approximately 0.098 N and 1.2 N (1 N ≈ 102 g). In group teeth movements, these forces can reach 250–300 g (2.45–2.94 N) [11].

Regarding the brands under study, we chose Fatscrew^®^ (Fts) MIs [6]; their monetary value is high due to the characteristics of the material used (groups G1, G2, G3 and G4). We decided to compare these with white label (MB) MIs, available at one of the world’s most popular websites, eBay^®^, because these were much more affordable than the other brands (groups G5, G6, G7 and G8). Both brands used a titanium alloy Ti6AI4V (grade V). Ti6AI4V MIs have better mechanical properties due to their small diameter, corrosion resistance, ease of removal and ease of manufacture compared to type IV, thus improving the performance of MIs [25,27].

When comparing the two brands, there were statistically significant differences in both loss of stability and fracture. In both cases, the Fts brand (groups G1, G2, G3 and G4) showed greater average strength compared to MB (groups G5, G6, G7 and G8).

The interaction between brand and diameter was statistically significant for both brands in terms of loss of primary stability and fracture. In relation to the loss of primary stability in the Fts brand, MIs with a diameter of 2.0 mm (groups G3 and G4) showed a higher average force compared to MIs with a diameter of only 1.6 mm (groups G1 and G2). The null hypothesis that there is no influence of the diameter of the Fatscrew MIs on resistance to bending force is therefore rejected.

In terms of the loss of primary stability and the fracture of the white band (MB) MIs, the highest average force corresponded to the 2.0 mm MIs (groups G7 and G8). Therefore, the null hypothesis that the diameters of MB MIs have no influence on resistance to bending force is rejected. These results are not in line with those regarding the main effects (when only the brands are compared to each other). The reason for this was related to the effect on the MB with a smaller diameter, 1.6 mm, where the adjusted average was much lower compared to the Fts brand with a 1.6 mm diameter.

These results are similar to those from studies analysing the influence of diameter on the loss of primary stability [7,10,24]. Wilmes et al. [7] observed that MI diameter was significantly associated with stability through torsional force. Barros et al. [10] concluded that flexural strength was significantly influenced by diameter, which was also confirmed by Haghigh et al. [24].

The interaction between brand and placement was statistically significant. The difference between the brands was statistically significant when there were two bone insertions, with a greater force being observed for the loss of primary stability as well as for the fracture of the Fts MIs (groups G2 and G4) compared to the MB MIs (groups G6 and G8). Therefore, the null hypothesis is rejected, as there were no differences between the MIs of the different brands in terms of resistance to bending force when placed twice in the bone.

When placed once, there were no statistically significant differences between the brands in terms of loss of primary stability. Therefore, the null hypothesis that there is no difference between the MIs of the different brands in relation to bending force after one placement in the bone is accepted. As for the fracture force of the MIs, there were statistically significant differences between the two brands when inserted once in the bone.

For the interaction effects of the three levels, brand, diameter and placement, no significant differences were found in the loss of primary stability. The highest average force was found in the white brand with a 2.0 mm diameter MIs placed both once and twice in the bone. The worst result, with a large difference in loss of stability force, was given by the 1.6 mm MB MIs placed once and twice in the bone.

As for the fracture of the MIs, the interaction effects of the three levels, brand, diameter and placement, were found to be significantly different. The highest average force was found in the white band with 2.0 mm diameter MIs placed both once and twice in the bone and with the 2.0 mm diameter Fts MIs placed once and twice in the bone. The worst result, with a large difference in fracture strength, was given by the 1.6 mm MB MIs placed once and twice.

Some differences were observed in the fracturing of the MIs during the laboratory tests. The only MIs that fractured in the MI head area were the 2.0 mm diameter MBs placed twice in the bone (Group G8). In this group, four of five MIs fractured in the head area, and another fractured in the spiral area. One hypothesis is that these are probably made of a less resistant material compared to the Fts MIs. Also, as they were reattached to the bone, both the neck and the head of the MI may have become more fragile due to the placement strength and, therefore, tended to fracture more. The remaining MB MIs fractured at the level of the coils (internal fracture).

In the Fts MIs, some showed internal fracture and others permanent deformation. From these results, we can conclude that one of the major differences between the two brands studied relates to the material the MI is made of. The Fts are made of a stronger and more elastic material than the MB, even though both are made of titanium alloy.

Although the results indicate a significant difference in the brand to be used, both demonstrated the capacity for temporary anchorage in orthodontic treatment, regardless of the type of movement to be performed. Therefore, we can use any of the brands studied, but it is important to note that the Fatscrew brand shows superior resistance to bending force and a significant difference in terms of fracture. Considering the financial aspect, it is important to note that the Fatscrew brand, although more expensive, offers superior resistance and is more elastic. Therefore, when deciding between the two options, it is crucial to weigh the cost against durability and the ability to withstand the necessary force.

While the cost of Fatscrew^®^ may initially be higher, its superior strength can result in savings in the long run, avoiding additional replacement or maintenance costs.

It is important to note that one of the limitations of our research was the fact that it was an in vitro study, and although the artificial bone used, Sawbones^®^, was the most suitable for testing with standardised models in order to achieve consistent results, it is not fully equivalent to natural bone due to variations in chemical composition and physical integrity. In addition, it is crucial to consider that bone composition differs depending on the individual, with significant variations possible. It is also necessary to recognise the limitations of the study environment, which, because it does not faithfully replicate the oral cavity, can influence the performance of the MIs, especially the MBs, which showed less resistance. One of the disadvantages is that humans do not have the same characteristics in terms of the % of cancellous bone and cortical bone in the jawbone constitution, and these results can differ from individual to individual. If the patient has periodontitis, the oral environment may become more acidic, leading to an increase in chemical reactivity, which may cause some modification in the MIs used [28]. One possible way to improve this resistance is to treat the surface of the MI with 2-methacryloyloxyethyl phosphocholine (MPC), which was proven in the study by Chen et al., who concluded that MPC is a favourable tool for preventing infectious diseases and inhibiting microbial activity in the implants studied [29]. Another relevant aspect is the impact of the sterilisation of the MIs, which can affect their effectiveness and is an additional factor to be considered when interpreting the results.

## 5. Conclusions

Considering the results achieved and in accordance with the methodology described in this study, we can conclude that, although the results show significant differences in the brands used, both demonstrated the capacity for temporary anchorage. However, it is important to note that the Fatscrew brand and a 2.0 mm diameter endow greater resistance to bending force. We can also conclude that MIs inserted once or twice did not lead to a statistically significant effect, so we can conclude that the operator can insert the MIs twice in the same patient without leading to a loss of stability. However, the Fatscrew MIs placed twice yielded better results than the white brand MIs. The only MIs that fractured in the MI head area were the 2.0 mm diameter MB MIs placed twice in the bone, showing significant differences in fracture between brands. We can conclude that the use of 1.6 mm diameter MIs is advisable, as they support the optimum force for orthodontic dental movement, as well as reducing the risk of injury to anatomical structures, especially adjacent teeth roots.

## Figures and Tables

**Table 1:** Main effects on force (N) at the loss of primary stability.

	Adjusted Means (SE)	Main Effects
Brand		
Fatscrew (Fts)	151.92 (4.61)	F_(1,32)_ = 9.06, *p* = 0.005, η^2^_g_ = 0.22
White label	132.31 (4.61)	
Insertions		
1	137.92 (4.61)	F_(1,32)_ = 1.67, *p* = 0.207, η^2^_g_ = 0.05
2	146.31 (4.61)	
Diameter		
1.6 mm	95.85 (4.61)	F_(1,32)_ = 201.59, *p* < 0.001, η^2^_g_ = 0.86
2.0 mm	188.38 (4.61)	

SE, standard error.

**Table 2:** Two-way effects on force (N) at loss of primary stability (brand and insertions × diameter).

	Diameter = 1.6 mm	Diameter = 2.0 mm	2-Way Effects
Brand			
Fatscrew (Fts)	125.09 (6.52)	178.75 (6.52)	F_(1,32)_ = 35.57, *p* < 0.001, η^2^_g_ = 0.53
White label	66.61 (6.52)	198.01 (6.52)	
Insertions			
1	92.54 (6.52)	183.31 (6.52)	F_(1,32)_ = 0.07, *p* = 0.788, η^2^_g_ = 0.00
2	99.16 (6.52)	193.46 (6.52)	

Results are presented as adjusted means and standard errors.

**Table 3:** Two-way effects on force (N) at loss of primary stability (brand × insertions).

Brand	Insertions = 1	Insertions = 2	2-Way Effects
Fatscrew (Fts)	140.26 (6.52)	163.58 (6.52)	F_(1,32)_ = 5.25, *p* = 0.029, η^2^_g_ = 0.14
White label	135.58 (6.52)	129.04 (6.52)	

Results are presented as adjusted means and standard errors.

**Table 4:** Three-way effects on force (N) at loss of primary stability (brand × insertions × diameter).

Brand	Diameter	Insertions	AdjM (SE)	3-Way Effects
Fatscrew (Fts)	1.6 mm	1	112.48 (9.22)	F_(1,32)_ = 0.32, *p* = 0.578, η^2^_g_ = 0.01
		2	137.70 (9.22)	
	2.0 mm	1	168.04 (9.22)	
		2	189.46 (9.22)	
White label	1.6 mm	1	72.60 (9.22)	
		2	60.62 (9.22)	
	2.0 mm	1	198.57 (9.22)	
		2	197.46 (9.22)	

Results are presented as adjusted means and standard errors.

**Table 5:** Main effects on force (N) at fracture.

	Adjusted Means (SE)	Main Effects
Brand		
Fatscrew (Fts)	170.11 (3.48)	F_(1,32)_ = 9.39, *p* < 0.001, η^2^_g_ = 0.50
White label	142.58 (3.48)	
Insertions		
1	155.04 (3.48)	F_(1,32)_ = 0.28, *p* = 0.598, η^2^_g_ = 0.01
2	157.66 (3.48)	
Diameter		
1.6 mm	107.06 (3.48)	F_(1,32)_ = 402.42, *p* < 0.001, η^2^_g_ = 0.93
2.0 mm	205.64 (3.48)	

SE, standard errors.

**Table 6:** Two-way effects on force (N) at fracture (brand and insertions *×* diameter).

	Diameter = 1.6 mm	Diameter = 2.0 mm	2-Way Effects
Brand			
Fatscrew (Fts)	138.08 (4.91)	202.15 (4.91)	F_(1,32)_ = 49.32, *p* < 0.001, η^2^_g_ = 0.61
White label	76.03 (4.91)	209.13 (4.91)	
Insertions			
1	106.42 (4.91)	203.66 (4.91)	F_(1,32)_ = 0.07, *p* = 0.787, η^2^_g_ = 0.00
2	107.70 (4.91)	207.62 (4.91)	

Results are presented as adjusted means and standard errors.

**Table 7:** Two-way effects on force (N) at fracture (brand *×* insertions).

Brand	Insertions = 1	Insertions = 2	2-Way Effects
Fatscrew (Fts)	162.36 (4.91)	177.76 (4.91)	F_(1,32)_ = 6.87, *p* = 0.013, η^2^_g_ = 0.18
White label	147.71 (4.91)	137.45 (4.91)	

Results are presented as adjusted means and standard errors.

**Table 8:** Three-way effects on force (N) at fracture (brand *×* insertions *×* diameter).

Brand	Diameter	Insertions	AdjM (SE)	3-Way Effects
Fatscrew (Fts)	1.6 mm	1	125.93 (6.95)	F_(1,32)_ = 6.87, *p* = 0.013, η^2^_g_ = 0.18
		2	150.23 (6.95)	
	2.0 mm	1	198.80 (6.95)	
		2	205.50 (6.95)	
White label	1.6 mm	1	86.90 (6.95)	
		2	65.16 (6.95)	
	2.0 mm	1	208.52 (6.95)	
		2	209.73 (6.95)	

Results are presented as adjusted means and standard errors.

## Data Availability

Data that support this study’s findings are available from the corresponding author upon request.
